# Proton stereotactic centralized ablative radiation therapy for treating bulky tumor: a treatment plan study

**DOI:** 10.3389/fonc.2025.1474327

**Published:** 2025-03-20

**Authors:** Tengxiang Li, Xinsen Yao, Ruimin He, Xian Xue, Shuai Wang, Jinhu Chen, Qingtao Qiu, Yong Yin, Quan Tang

**Affiliations:** ^1^ School of Nuclear Science and Technology, University of South China, Hengyang, China; ^2^ Department of Radiation Physics, Shandong Cancer Hospital and Institute, Shandong First Medical University and Shandong Academy of Medical Sciences, Jinan, China; ^3^ Department of Radiotherapy Center, Chenzhou NO.1 People’s Hospital, Chenzhou, China; ^4^ Department of Oncology, The Second Affiliated Hospital, Hengyang Medical School, University of South China, Hengyang, China; ^5^ Institute of Radiation Protection and Nuclear Safety Medicine, Chinese Center for Disease Control and Prevention, Beijing, China; ^6^ Department of Radiation Oncology, The First Affiliated Hospital of USTC, Division of Life Sciences and Medicine, University of Science and Technology of China, Hefei, Anhui, China; ^7^ University of Science and Technology of China (USTC), School of Nuclear Science and Technology (SNST), Hefei, Anhui, China

**Keywords:** stereotactic, centralized/core ablative, proton, bulky tumor, radiation therapy

## Abstract

**Objective:**

Stereotactic centralized/core ablative radiation therapy (SCART) is a novel radiotherapy approach. This study investigates the potential benefits of proton-based SCART (pSCART) by leveraging the dosimetric advantages of protons and integrating them with the SCART technique.

**Methods:**

Five clinical cases previously treated with conventional proton therapy were selected for this study. The pSCART plans utilized a relative biological effectiveness (RBE) prescription dose of 24 Gy (RBE) × 3 fractions, with each plan consisting of three to five fields. The prescribed dose for the CyberKnife SCART was the highest value meeting the organs-at-risk (OARs) dose limits and the tumor edge dose limits. The dose distributions of the CyberKnife-based SCART and pSCART plans were compared using five criteria: i) prescription dose; ii) 80% prescription dose volume, targets coverage at 80% and 20% dose levels, and the 80%/20% ratio; iii) volume receiving >5 Gy outside the tumor edge; iv) dose tolerance limits to OARs; and v) mean dose to OARs.

**Results:**

pSCART can deliver a higher prescription dose of 24 Gy × 3 fractions versus SCART’s 15 Gy × 2–3 fractions or 18 Gy × 2 fractions. Specifically, pSCART outperforms SCART in terms of the 80% prescription dose volume and 80% dose level coverage of stereotactic centralized/core target volumes (SCTV) achieving 69.77%–100.00% versus SCART’s 43.6%–99.5%. The 20% dose level coverage for gross target volume (GTV) is slightly lower for pSCART, achieving 88.96%–98.64% versus SCART’s 90.1%–99.9%. The maximum point dose outside the target volume is lower for pSCART at 4.58–6.19 Gy versus SCART’s 4.78–6.67 Gy; additionally, the V_5Gy_ at the tumor edge is significantly smaller for pSCART at 5.93–23.72 cm^3^ versus SCART’s 6.85–151.66 cm^3^. The average dose to most OARs in the pSCART plan is lower than in the SCART plan.

**Conclusions:**

This work provides initial insights into evaluating treatment plans for bulky tumors using pSCART. Compared to the CyberKnife SCART, pSCART generates significantly higher prescription doses and larger high-dose regions within the GTV while delivering lower doses at the tumor edge, enhancing normal tissue sparing.

## 1 Introduction

Patients with bulky tumors often have a worse prognosis and frequently receive only palliative treatments (1, 2). A novel treatment approach termed stereotactic centralized/core ablative radiation therapy (SCART) has been developed for managing bulky or metastatic tumors. SCART is based on the principles of spatially fractionated radiation therapy (SFRT) (3, 4) and builds upon stereotactic body radiotherapy (SBRT) to optimize the high-dose region within the tumor core, aiming to achieve enhanced ablative effects, particularly in areas harboring cancer stem cells or highly resistant progenitors (5–7). Concurrently, low-dose radiation to the tumor periphery may mitigate some of the immunosuppressive effects associated with radiation at the tumor edge (8).

In a phase I study, SCART administration for recurrent or metastatic bulky tumors demonstrated favorable tolerability and safety, allowing for dose escalation up to the maximum tolerated dose (MTD) of 24 Gy delivered in 3 fractions (4). While the radiobiological mechanisms underlying this increased therapeutic index remain incompletely described, potential contributors include dose volume effects (9), bystander-like effects (10), differential vascular effects (11), inflammation and immunomodulatory effects (5, 12), and cell migration (13–15). By integrating the strong spatial dose modulation capability of SCART with the superior dose deposition characteristics of protons, the concept of proton-based SCART (pSCART) was proposed. This study investigates whether pSCART can achieve higher prescription doses, larger central/core high-dose regions, and reduced doses at the tumor edge.

This work evaluates the potential benefits of pSCART for treating bulky tumors such as soft tissue sarcomas, hepatic metastases, and pancreatic cancer. Specifically, it assesses the ability of pSCART technology to achieve the desired dose distribution pattern of SCART while ensuring effective protection of organs at risk (OARs) under high prescription doses. This research aims to guide future evaluations of pSCART clinical trials and facilitate its clinical implementation.

## 2 Materials and methods

### 2.1 Clinical case selection

Five clinical cases were selected from the patient database of our institution (Shandong Cancer Hospital and Institute, China): three sarcomas located in different anatomical regions—above the clavicle (Case 1), at the back (Case 2), and in the lower limb (Case 5)—as well as a pancreatic tumor (Case 3) and hepatic metastases (Case 4). The selection criteria included the following:

Tumor location: Cases encompassed commonly targeted sites for SBRT.
Target size: Lesions were measurable by computed tomography (CT) imaging, with maximum axial dimensions exceeding 5 cm [consistent with SCART trial criteria (4)].

Proximity to critical OARs.

The selected five cases in this study are described in **Table 1**.

**Table 1:** Tumor characteristics and treatment parameters.

	The site of tumor	Type	GTV vol. (cm^3^)	SCTV vol. (cm^3^)	SCTV vs. GTV
Case 1	Left supraclavicular	Sarcoma	80.76	3.71	4.59%
Case 2	Right back	Sarcoma	996.8	46.37	4.59%
Case 3	Abdomen	Pancreatic cancer	367.83	16.65	4.53%
Case 4	Abdomen	Hepatic metastases	694.46	33.84	4.87%
Case 5	Right leg	Sarcoma	559.33	26.16	4.68%

GTV, gross target volume; SCTV, stereotactic centralized/core target volumes.

All procedures adhered to institutional review board guidelines, and informed consent was waived for this retrospective planning study using fully anonymized CT datasets.

### 2.2 Treatment planning

#### CT acquisition protocol

Simulations were conducted using a SOMATOM Drive dual-source CT scanner (Siemens Healthineers AG, Forchheim, Germany) with a slice thickness of 1 mm. The scanning range extended at least 15 cm beyond the tumor margins in all directions.

CyberKnife-based SCART planning:

Delivery system: CyberKnife M6 C0521 (Accuray Inc., Sunnyvale, CA, USA);
Dose algorithm: finite-size pencil beam with side scatter (FSPB+); and
Dose recalculations: Monte Carlo recalculations with 1% statistical uncertainty.

#### Proton pSCART planning

Field set-up: Three to five fields were used, with a gantry angle interval of approximately 30° between adjacent fields. Non-coplanar fields can be adopted if necessary. Fields were positioned at appropriate angles near the tumor to avoid regions with significant density changes and the edge of the treatment couch. Each plan was completed by an experienced physicist from this institution who specializes in proton therapy.
Treatment planning system: RayStation V12.0.100.0 (RaySearch Laboratories, Stockholm, Sweden);
Beam model: Probeam™ TR3 proton therapy system (Varian Medical Systems, Palo Alto, CA, USA);
Dose algorithm: IonMonteCarlo with 1% statistical uncertainty;
Relative biological effectiveness (RBE): RBE = 1.1 per ICRU Report 78 recommendations (16); and
Robust optimization: accounts for ±3.5% range uncertainty and ±5 mm set-up errors.

#### Prescription dose schemes

The dose escalation framework was derived from the MTD trial of Dr. Yang et al. (4). The prescribed dose was based on the maximum dose to the gross target volume (GTV), as established in preclinical trials. The prescription dose was normalized to the maximum dose point within the treatment plan.

#### CyberKnife SCART

Prescription dose range: 15 Gy × 1 fraction to 24 Gy × 3 fractions;
Prescription dose determination: The fraction and prescribed dose of the SCART plan were determined based on OARs dose limits and the volumetric spillage (V_5Gy_ in non-target tissue per fraction).

#### Proton pSCART

Prescription dose: The highest dose of 24 Gy (RBE) × 3 fractions was utilized as determined in the MTD trial of Dr. Yang et al.

### 2.3 Dosimetric properties and metrics

The evaluation and comparison of SCART and pSCART considered three main points:

1. The target coverage in SCART and pSCART. SCART and pSCART plans considering an 80% prescribed dose coverage of stereotactic centralized/core target volumes (SCTV), with V_80_% ≥ 95%, and a 20% prescribed dose coverage of the GTV, with V_20_% ≥ 90%. Additionally, for SCART treatments, the ratio between the 80% and 20% prescribed doses (V_80_%/V_20_%) was computed, ensuring that this ratio exceeded 4.5% (4).
2. The extra-target dose distribution was quantified using two primary dosimetric indices: V_5Gy_, the volume of non-target tissue receiving upper 5 Gy per fraction; and high-dose limit, the maximum dose to a specified volume outside the GTV. Both parameters were minimized through iterative optimization cycles.
3. The dose tolerance limits for OARs. For the SCART and pSCART plans, the dose distribution was similar to that of SBRT with an exceptionally high central dose. Consequently, existing SBRT dose limit values for OARs were employed to evaluate the pSCART plan. For normal organs like the bone, there were no strict and effective assessment indicators for tolerance to high-dose proton irradiation with fewer fractions. Therefore, dose limits for SCART were based on standard irradiation schemes (2 Gy per fraction). The normalized total dose (NTD) at 2 Gy-fractions, $NTD_{2.0}$ , was computed using **Equation 1**.

(1) $$NTD_{2.0}=n\cdot d\left(1+\frac{d}{\alpha /\beta }\right){\left(1+\frac{2Gy}{\alpha /\beta }\right)}^{-1}.$$

The average dose to the femoral bone structures was converted to $NTD_{2.0}$ , as the risk of radiation-induced complications was proportional to the mean dose of the organ (17). The biologically effective dose (BED) to OARs was computed using **Equation 2**.


(2) $$BED=n\cdot d\left(1+\frac{d}{\alpha /\beta }\right),$$

where the *α*/*β* values follow ICRU Report 91 recommendations (18). Radiation dose fractionation data for fractures in patients suggest an alpha/beta (*α/β*) ratio in the range of 1.8–2.8 Gy (19).

## 3 Results

The corresponding prescribed doses for the CyberKnife SCART and pSCART plans can be found in **Table 2**.

**Table 2:** Treatment plans evaluated in this study.

	Equipment	Technique	Fractions	Prescribed dose (Gy)	Gantry angle (°)
Case 1	CyberKnife	SCART	2	36.0	Defined by TPS
	Proton	pSCART	3	72.0 (RBE)	330/0/70/135
Case 2	CyberKnife	SCART	2	30.0	Defined by TPS
	Proton	pSCART	3	72.0 (RBE)	180/270/305/340
Case 3	CyberKnife	SCART	2	30.0	Defined by TPS
	Proton	pSCART	3	72.0 (RBE)	280/310/340/10/40
Case 4	CyberKnife	SCART	3	45.0	Defined by TPS
	Proton	pSCART	3	72.0 (RBE)	180/260/300/320
Case 5	CyberKnife	SCART	3	45.0	Defined by TPS
	Proton	pSCART	3	72.0 (RBE)	180/235/280/330

The CyberKnife selects the best field combination from 3,600 discrete incident angles in the treatment planning system (TPS), enabling precise non-coplanar dose deposition. Proton therapy plans predominantly utilize coplanar field configurations; however, for specific anatomical sites (e.g., Case 1), non-coplanar fields are necessary to enhance dose conformity and sparing of organs at risk (OARs). In Case 1, a gantry angle of 135°combined with a couch angle of 315° was utilized.

SCART, stereotactic centralized/core ablative radiation therapy; pSCART, proton-based SCART; RBE, relative biological effectiveness.

The fractions and prescribed doses of the CyberKnife SCART treatment were based on the MTD ranges used in the previous phase I clinical trial conducted by Dr. Yang (4), with the extra-target dose not exceeding 5 Gy (i.e., the volume of non-target tissue exposed to more than 5 Gy should be minimized), and the OARs dose limits.

### 3.1 Dose distributions of SCART and pSCART

**Figure 1:**
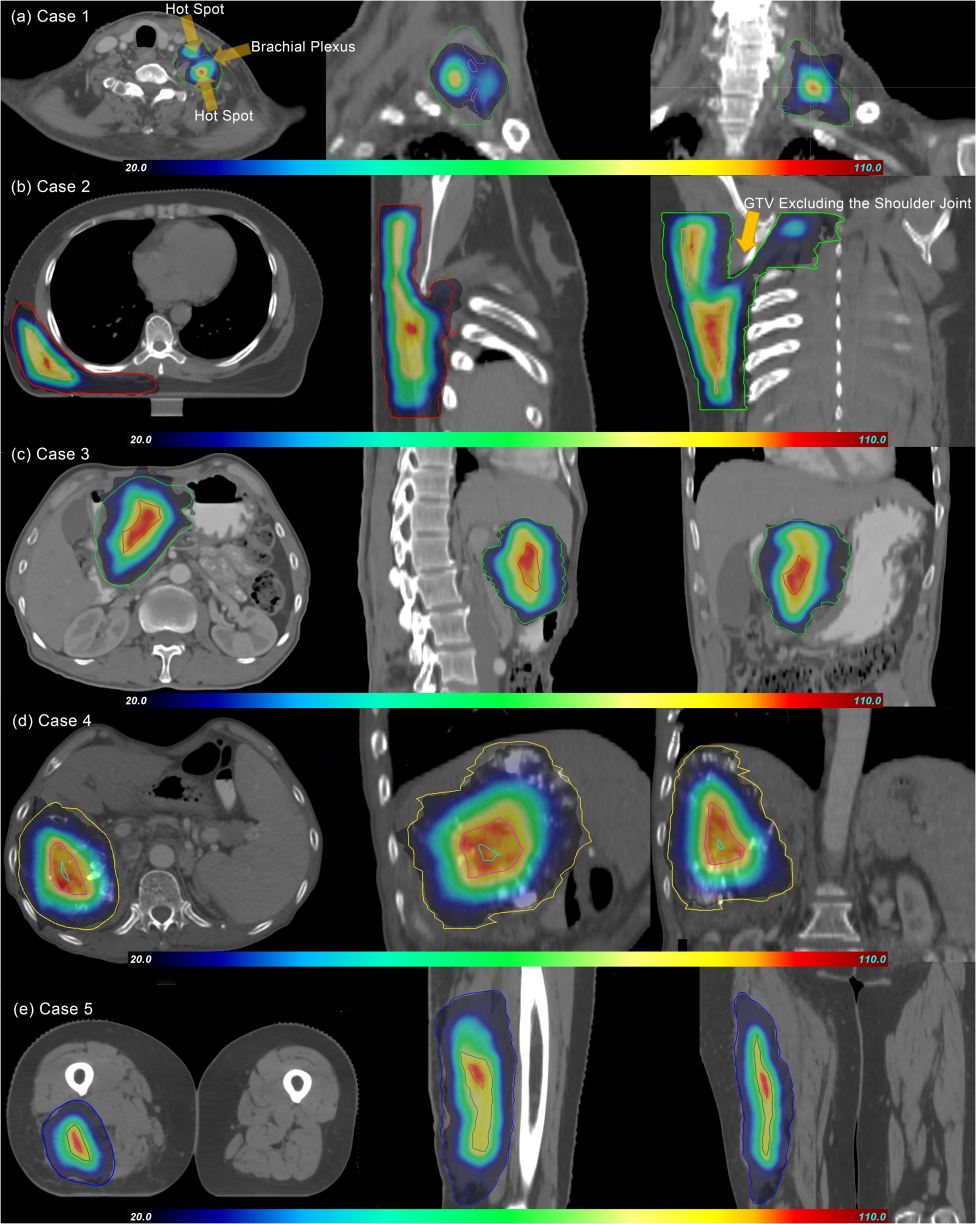
Dose distributions of pSCART treatments for the five cases. In the figure, GTV and SCTV are marked with contour lines of different colors. The dose gradient range is set from 20% to 110%, and the prescription dose is normalized with the maximum value of 100% as the benchmark. As shown in the figure, the dose volume spillage area outside GTV does not significantly exceed the 20% prescription dose threshold. Under the condition that functional imaging guidance is limited, the SCART technique theoretically should generate an SCTV with a regular geometric shape by uniformly shrinking GTV. However, in clinical practice, the areas overlapping with important serial organs like nerve plexuses (such as brachial plexus in Case 1), joint structures (such as shoulder joint in Case 2), digestive tracts, and major blood vessels, which are organs at risk (OARs), need to be excluded from SCTV to form the “SCTV-OARs” modified target area. This anatomical structure exclusion operation leads to the irregularity of SCTV in the pSCART plan. The research results show that the proton plan with active beam modulation is superior to the CyberKnife SCART plan in terms of SCTV coverage and conformity. This result is also verified by the “80% SCTV coverage” data in **Table 3**. SCART, stereotactic centralized/core ablative radiation therapy; pSCART, proton-based SCART; GTV, gross target volume; SCTV, stereotactic centralized/core target volumes.

In the pSCART plans, for Cases 1 and 2, where the organs at risk were located within or near the target volume with suboptimal dose gradients, inferior conformity was observed. In contrast, superior conformity was demonstrated in the remaining cases. The dose distributions of the pSCART plans for the five evaluated cases are illustrated in **Figure 1**.

**Figure 2:**
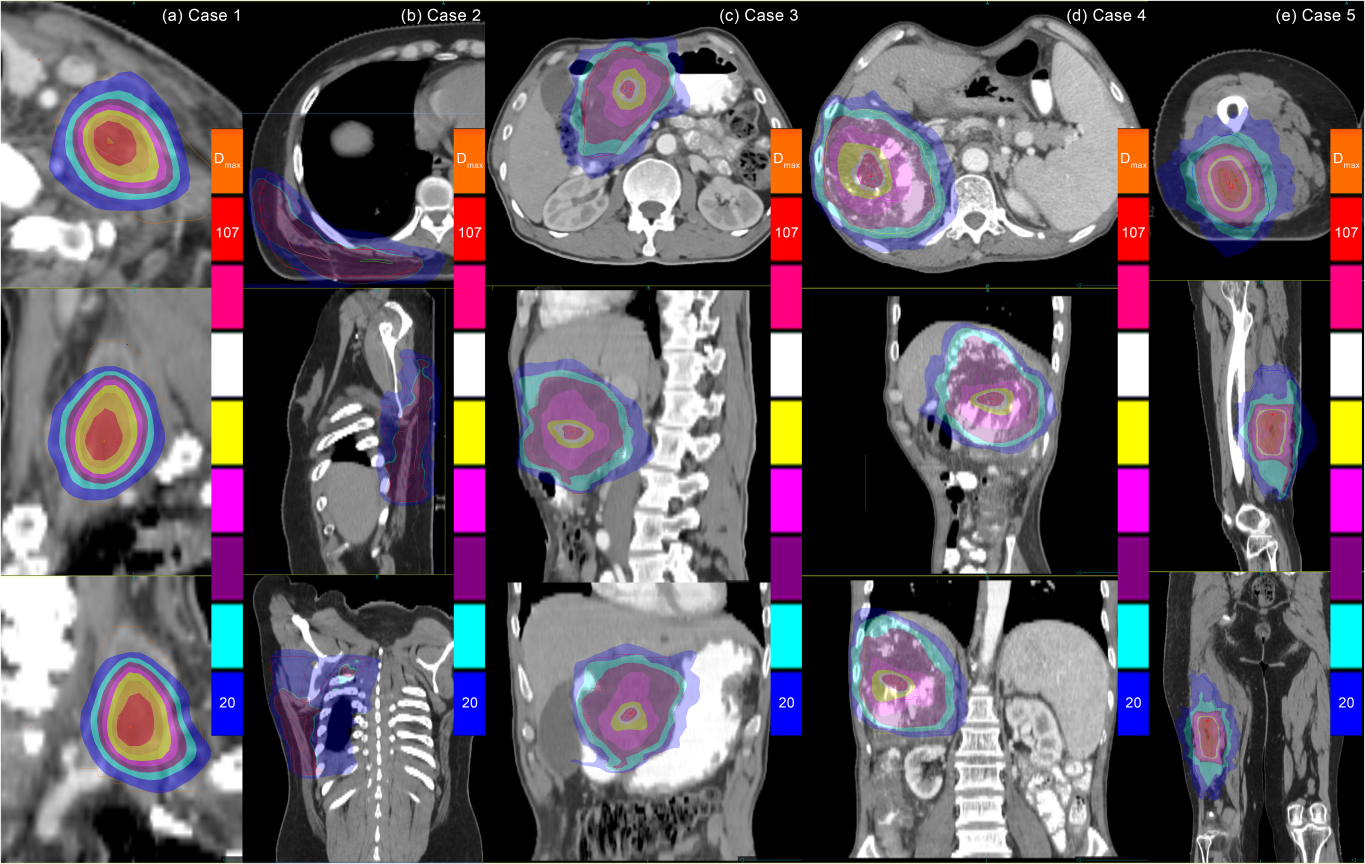
Dose distributions of CyberKnife SCART treatments for the five cases. Note: Dosimetric normalization to D_max_ resulted in gradient ranges of 20%–107%, with significant dose heterogeneity observed in GTV. While CyberKnife demonstrates enhanced plan design proficiency in meeting SCART clinical criteria, its inherent physical limitations hinder effective modulation of steep dose gradients between the high-dose regions of the SCTV and the low-dose regions adjacent to organs at risk (OARs). Quantitative analysis reveals compromised dosimetric performance for irregular target geometries (Cases 2 and 5) compared to regular-shaped targets. SCART, stereotactic centralized/core ablative radiation therapy; GTV, gross target volume.

The target dose distribution for the SCART plan delivered by the CyberKnife is presented in **Figure 2**.

**Figure 3:**
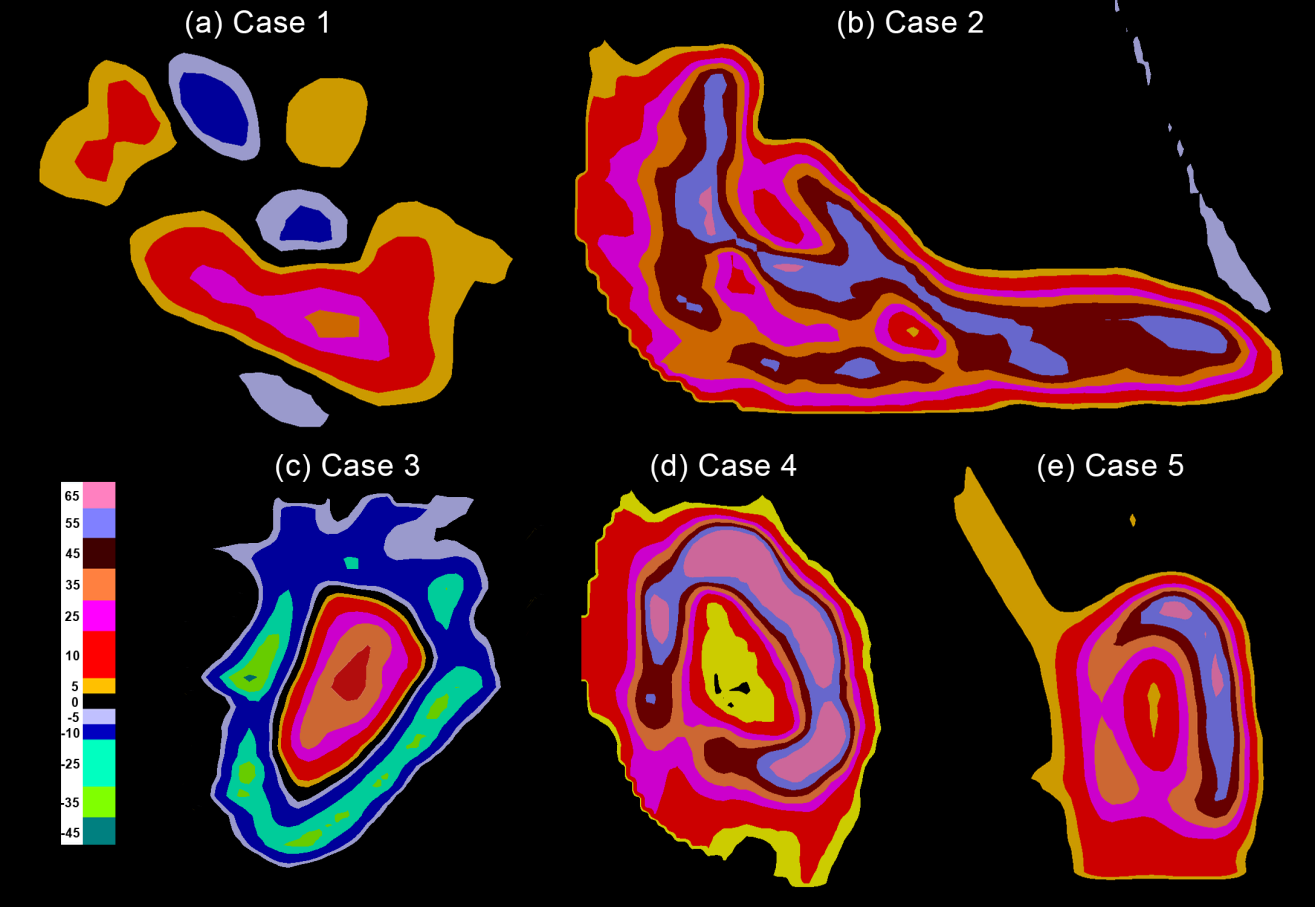
The target dose differences between pSCART and uniform dose proton plans. Note: Compared with proton therapy plans that employ uniform dose distribution, the pSCART technique achieves a dose escalation effect by increasing the dose at the center of the target volume. Dosimetric comparative analysis reveals that the pSCART plan exhibits a characteristic asymmetric dose distribution relative to the patient’s original treatment plan. This spatial dose heterogeneity may enhance the biological response of SFRT through the radiation bystander effect; however, its dose–effect relationship requires systematic validation through animal and clinical trials. SCART, stereotactic centralized/core ablative radiation therapy; pSCART, proton-based SCART; SFRT, spatially fractionated radiation therapy.

Compared to conventional proton therapy plans, the pSCART plans require the generation of exceptionally high dose hot spots within the target area, resulting in a rapid dose falloff around the central region of the target volume. In Case 1, more stringent constraints were applied in the pSCART plan to protect the brachial plexus, leading to the formation of two distinct hot regions on either side of the brachial plexus. One region exhibits a maximum dose equivalent to the prescribed dose, while the other shows a lower dose. The differences in target dose distribution between pSCART and conventional proton therapy are illustrated in **Figure 3**.

### 3.2 Target coverage

The comparison of dosimetric indices defining target volume and coverage between SCART and pSCART is illustrated in **Table 3**.

**Table 3:** Dosimetric indexes of the target coverage in the CyberKnife SCART and proton-based SCART.

	Equipment	Technique	Vol. of 80% (cm^3^)	80%/20%	80% cover of GTV	20% cover of GTV	80% cover of SCTV
Case 1	CyberKnife	SCART	7.22	6.06%	8.9%	90.1%	97.2%
	Proton	pSCART	0.46	0.74%	7.66%	94.16%	96.24%
Case 2	CyberKnife	SCART	31.11	1.59%	3.1%	96.6%	43.6%
	Proton	pSCART	48.28	5.28%	5.38%	91.53%	69.77%
Case 3	CyberKnife	SCART	15.44	2.07%	4.2%	99.9%	99.5%
	Proton	pSCART	39.00	11.11%	8.48%	92.86%	100.00%
Case 4	CyberKnife	SCART	38.09	5.55%	5.5%	98.4%	66.6%
	Proton	pSCART	59.05	9.95%	9.23%	88.96%	100.00%
Case 5	CyberKnife	SCART	67.57	5.37%	12.0%	98.4%	74.2%
	Proton	pSCART	40.10	7.17%	7.6%	98.64%	97.13%

The shaded area in gray indicates that the results of pSCART are lower in comparison.

GTV, gross target volume; SCTV, stereotactic centralized/core target volumes; SCART, stereotactic centralized/core ablative radiation therapy; pSCART, proton-based SCART.

In all instances except Case 1, the ratio of the 80% to 20% prescribed dose volume in the pSCART plan surpassed that in the SCART plan. According to the results in **Table 3**, the coverage of 80% of the prescribed dose to the GTV in all pSCART plans exceeded 4.5%. For Cases 2 to 5, the ratio of 80% to 20% of the prescribed dose was not only higher than 4.5% but also significantly greater than that observed in the CyberKnife SCART plans. This suggests that pSCART demonstrates a superior capability in generating central/core high-dose regions compared to the CyberKnife SCART and the linear accelerator with Volumetric Modulated Arc Therapy (VMAT) or Intensity-Modulated Radiotherapy (IMRT) technology used in the MTD trial of Dr. Yang et al.

### 3.3 Limit of high dose at tumor edge

The study compared dosimetric parameters for the generation of high-dose regions outside the target volume in the CyberKnife SCART and pSCART plans. The detailed parameters include two key indicators: volume and dose. Specifically, these parameters encompass the volume receiving a dose exceeding 5 Gy per fraction outside the target area, and the corresponding dose value for this high-dose volume should be minimized.

In the pSCART plans, these parameters were superior to those in the CyberKnife SCART plans, as illustrated in **Table 4**.

**Table 4:** Dosimetric indices for high dose at tumor edge in SCART and pSCART.

	Equipment	Technique	V_5Gy_ (cm^3^)	Gy/fractions
Case 1	CyberKnife	SCART	6.85	4.78
	Proton	pSCART	6.75	4.71
Case 2	CyberKnife	SCART	151.66	6.67
	Proton	pSCART	23.72	5.19
Case 3	CyberKnife	SCART	66.98	6.17
	Proton	pSCART	17.53	5.17
Case 4	CyberKnife	SCART	106.15	6.19
	Proton	pSCART	5.93	4.58
Case 5	CyberKnife	SCART	112.08	6.12
	Proton	pSCART	19.38	5.13

The results marked in gray indicate that the dose outside the target volume is less than 5 Gy/fraction or that the V_5Gy_ is less than 20 cm^3^.

SCART, stereotactic centralized/core ablative radiation therapy; pSCART, proton-based SCART.

The prescribed dose in the CyberKnife SCART plan refers to the dose range from 15 Gy × 1 fraction to 24 Gy × 3 fractions and aims to minimize the high-dose region outside the target volume. For Case 1, both the CyberKnife SCART and pSCART plans achieved a smaller V_5Gy_ and lower dose outside the target area. Additionally, the pSCART plan for Case 4 also demonstrated this advantage. However, the remaining cases did not meet the requirement of maintaining doses outside the target area below 5 Gy per fraction, although the pSCART plan demonstrates better performance. Furthermore, the quality of the plan can be evaluated by restricting and assessing the volume size exceeding 5 Gy. In the pSCART plan, it was possible to achieve V_5Gy_ less than 20 cm^3^, or close to 20 cm^3^.

### 3.4 Assessment of dose tolerance limits

In Case 1, the maximum dose to the left brachial plexus was 27.25 Gy, exceeding the dose limits. However, only 0.78 cm^3^ received a dose of 20.4 Gy, meeting the requirement of at most 3 cm^3^ at this dose level.

In Case 2, for the right lung, the spared volume from doses of 12.40 Gy was 1,410.76 cm^3^; for all lungs, the spared volume was 2,490.4 cm^3^ at 12.40 Gy and 2,489.33 cm^3^ at 11.60 Gy, meeting the requirements of 1,000 and 1,500 cm^3^, respectively. For the spinal cord, the maximum dose was 9.27 Gy, with no volume receiving doses of 18 or 12.3 Gy, while only 0.35 cm^3^ received a dose of 8.63 Gy.

In Case 3, the duodenum received a maximum dose of 18.6 Gy, with 0.58 cm^3^ at 16.5 Gy and 11.56 cm^3^ at 11.4 Gy. For the right kidney, 66% of its volume received a dose of 0.48 Gy, with no volume receiving 16 Gy. The left kidney received no dose. For the liver, there was no volume receiving a dose of 19.2 Gy, indicating a spared volume of 1,318.74 cm^3^ from doses of 19.2 Gy. For the stomach, the maximum dose was 15.26 Gy, with only 0.18 cm^3^ receiving a dose of 16.5 Gy.

In Case 4, the duodenum showed no volume receiving a dose of 16.5 Gy, with only 0.59 cm^3^ at 11.4 Gy and a maximum dose of 12.23 Gy. For the liver, the spared volume from doses of 19.2 Gy was 1,059.39 cm^3^, and the maximum dose was 14.8 Gy.

In Case 5, the bone received a maximum dose of 9.64 Gy and a mean dose of 0.7 Gy. Using Formulas 1 and 2, the calculated $NTD_{2.0}$ (Equation 1) for the bone ranged from 10.46 to 10.88 Gy, significantly lower than the threshold of 40 Gy associated with femoral fracture (17).

**Figure 4:**
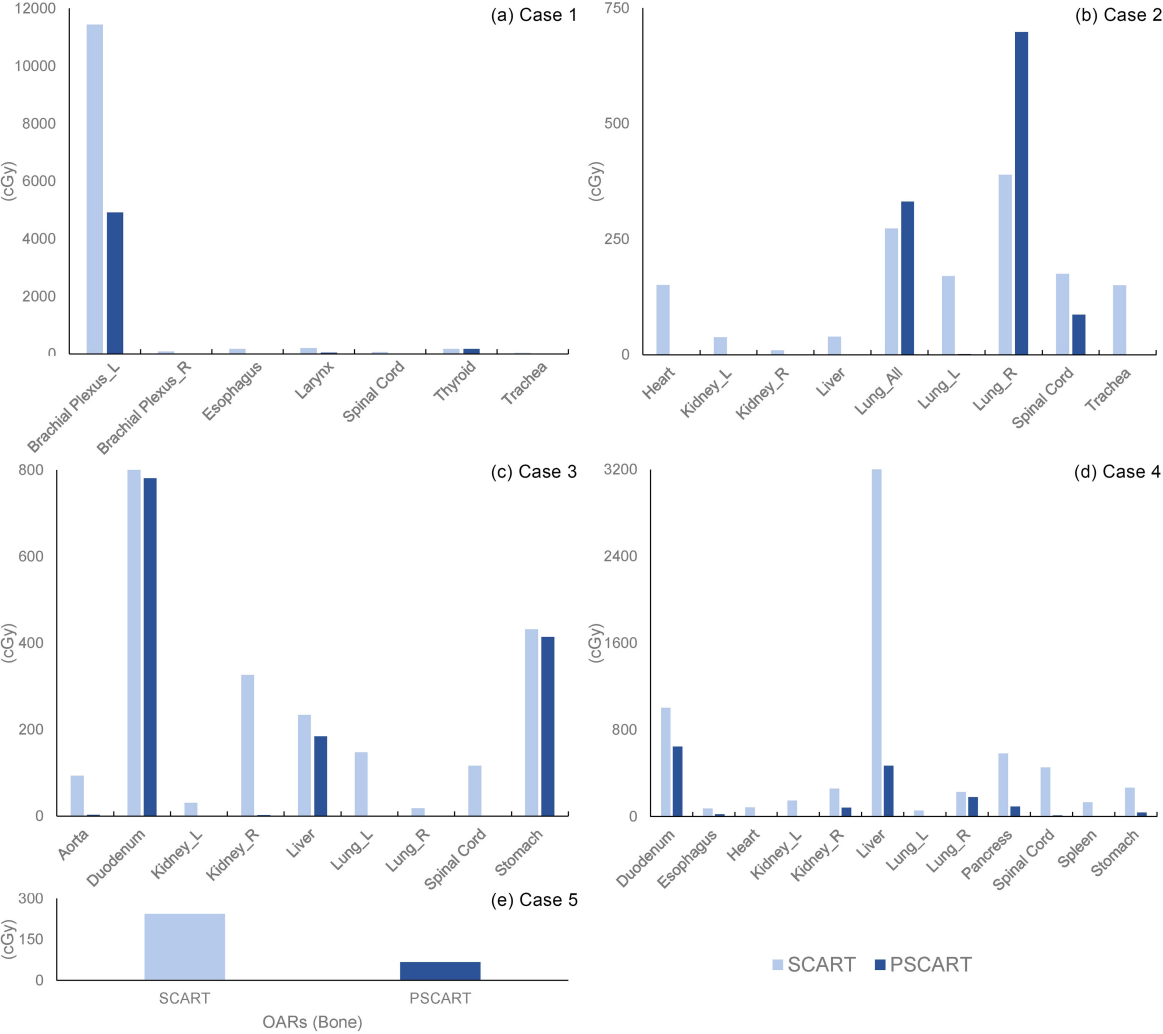
The average dosage of OARs in the CyberKnife SCART and pSCART plans. Note: The figure comparing the mean dose to OARs highlights the differences between SCART and pSCART plans. Except for the affected lung and total lungs in Case 2, the mean dose for all other cases in pSCART was lower than that in the SCART, with some OARs receiving no significant dose. It is important to note that mean dose has limitations in assessing serial organs, as it may not fully capture the potential damage to critical serial organs. Therefore, in the practical application of pSCART, it is recommended to emphasize the dose limits established for existing SBRT protocols. OARs, organs at risk; SCART, stereotactic centralized/core ablative radiation therapy; pSCART, proton-based SCART; SBRT, stereotactic body radiotherapy.

The mean dose to the target to OARs was calculated, and the mean BED (Equation 2) of OARs is illustrated in **Figures 4A–E**.

## 4 Discussion

Patients afflicted with bulky tumors often face a poor prognosis (3). However, enhancing local disease control in bulky tumors can significantly improve both overall survival (OS) and quality of life (QOL). The substantial tumor size poses a significant challenge for conventional radiotherapy (1), as it increases the risk of collateral damage to surrounding tissues. To mitigate this risk, alternative approaches such as SFRT are required (20). SFRT, including techniques like GRID (21, 22), LATTICE (23, 24), and Mini-beam therapy (25–27), has demonstrated benefits in treating bulky tumors (20). Excessive doses can induce tumor cell death through vascular damage and antitumor immunity (28). The generation of multiple “hot spots” (in the form of strips or islands) to encompass the entirety of bulky tumors may need to limit the size of individual hot spots. Consequently, the total volume covered by these hot spots remains comparatively small compared to the overall volume of the bulky tumors (4, 8).

SCART posits that increasing the proportion of the tumor’s central core receiving ablative doses can enhance biological effects (6). This approach triggers the bystander effect and abscopal effect. In SCART, distinct high-dose central regions, low-dose peripheries, and intermediate-dose areas coexist within the GTV. High doses can achieve effective ablation, and expanding the high-dose region can further enhance ablation efficacy. Meanwhile, administering a lower dose at the tumor margins helps protect adjacent normal tissues. Previous SCART studies have shown that the high-dose region typically accounts for approximately 4.5% of the GTV (4, 8, 29). Protons delivered via spot-scanning technology offer superior dose modulation capabilities and reduce side effects. Consequently, this paper proposes pSCART treatment and investigates whether pSCART can achieve higher prescription doses, larger central/core high-dose regions, and lower doses at the tumor edge.

This study provides initial insight into the feasibility of treating bulky tumors with pSCART. Results demonstrate that the pSCART can achieve a prescription dose of 24 Gy × 3 fractions at the central high-dose region, with high-dose regions occupying a volume significantly greater than 4.5% of the GTV. The coverage rate of the high-dose area to the SCTV was relatively high. The volume outside the target area receiving a dose exceeding 5 Gy was smaller, and the extent of the high-dose region outside the target area was lower. Based on the results of the dose outside the target area, the following dose limit conditions for pSCART could be initially proposed: the dose volume outside the target area exceeding 5 Gy per fraction should be less than 20 cm^3^.

Compared to CyberKnife-based SCART, pSCART can deliver a higher prescription dose and achieve comparable or superior tumor coverage while maintaining similar dose levels for OARs. The objective of SCART was not simply to administer a supra-high dose or a very large high-dose area but rather to adopt a balanced approach. Considering the protection of OARs as a premise, it may be necessary to adjust the prescription dose accordingly, potentially utilizing lower doses than those employed in this study. Compared to conventional proton radiotherapy, the pSCART plans deliver a high dose per fraction to the tumor (up to 72-Gy RBE total dose at the central region), particularly for targeting bulky lesions due to their ablative radiation dose nature. When not accounting for interplay effects and dose delivery errors due to respiratory motion, the pSCART plans based on active beam scanning (ABS) achieve more effective modulation of dose within the target volume. Daily patient set-up and tumor localization pose challenges due to organ movement relative to bony anatomy and inter-fraction organ deformation (30). Consequently, administering pSCART treatments in 3 fractions may reduce inter-fraction set-up uncertainties.

In the specific pSCART plans, the maximum dose received by the left brachial plexus in Case 1 and the dose of the volume at 11.4 Gy received by the duodenum in Case 3 did not meet the dose limit. Except for these two indicators, the remaining OARs in all cases conform to the prescribed dose limits for SBRT mentioned in AAPM TG101 reports (31). The brachial plexus in Case 1 was encompassed within the target volume, and the specific reduction of dose within the SCART target volume was unattainable. The pSCART plan yielded two distinct “hot spots” within the target volume, achieving the prescribed dose. In view of the lack of specific restrictions on bone dose in the existing dose limits for SBRT, in Case 5, the $NTD_{2.0}$ method was adopted for conversion to explore the possibility of comparison with dose limits that may lead to femoral fracture (17).

The assessment of toxicity to normal tissues anticipates that the Linear Quadratic (LQ) model will tend to overestimate the extent of damage, especially at doses exceeding 10 Gy per fraction (32). The approach to avoid femoral fracture by computing and evaluating the radiation dose to the bone using BED (Equation 1) and NTD (Equation 2) is conservative. Regarding the feasibility of delivering pSCART treatments in clinics, plans evaluated in this work are realizable using the experimental set-up already implemented for preclinical trials and treatment. The organ motion can be controlled as in current SBRT and common proton treatments. Further development of corresponding biological models and assessment parameters is still required for a more effective evaluation of the efficacy of pSCART.

## 5 Conclusions

This manuscript presents the first treatment plan evaluation of pSCART for treating sarcoma, pancreatic cancer, and liver cancer. Compared to the CyberKnife SCART, pSCART demonstrates superior dose prescription, larger central high-dose regions, and higher target coverage. Additionally, pSCART delivers lower doses at the tumor margins, reducing integral doses to OARs and enhancing normal tissue sparing. These advantages suggest an increased therapeutic index for bulky treatments. Phase I clinical trials are warranted to confirm these findings.

## Data Availability

The original contributions presented in the study are included in the article/supplementary material. Further inquiries can be directed to the corresponding authors.
